# Is perfectionism a risk factor for adolescent body dysmorphic symptoms? Evidence for a prospective association.

**DOI:** 10.1016/j.jocrd.2019.100445

**Published:** 2019-07

**Authors:** Georgina Krebs, Rachel Quinn, Amita Jassi

**Affiliations:** a King's College London, MRC Social, Genetic and Developmental Psychiatry Centre, Institute of Psychiatry, Psychology & Neuroscience, De Crespigny Park, London, United Kingdom; b National and Specialist OCD and Related Disorders Clinic for Young People, South London and Maudsley NHS Foundation Trust, London, UK; c King's College London, Department of Psychology, Institute of Psychiatry, Psychology & Neuroscience, De Crespigny Park, London, United Kingdom

**Keywords:** Perfectionism, Body dysmorphic disorder, Adolescence, Aetiology, Risk

## Abstract

The current study tested the hypothesis that perfectionism is a risk factor for the development of body dysmorphic disorder (BDD), as proposed by prevailing cognitive behavioural models. School students aged 14–16 years completed questionnaires 6 months apart (Time 1: *N* = 302; Time 2: *N* = 68) assessing perfectionism, BDD symptoms, and anxiety and depression. Robust regression models tested concurrent and prospective associations between perfectionism and BDD symptoms, with and without adjustment for coexisting anxiety and depression. Total perfectionism was positively associated with concurrent BDD symptoms, even when controlling for coexisting anxiety and depression. Moreover, total perfectionism predicted changes in BDD symptoms between Time 1 and Time 2. Examination of perfectionism subscales indicated that only self-oriented perfectionism, not socially-prescribed perfectionism, predicted BDD symptoms concurrently and prospectively while controlling for coexisting psychopathology. This study provides preliminary evidence for self-oriented perfectionism being a risk factor for the development of BDD in youth. If replicated, these findings could highlight the potential value of targeting self-oriented perfectionism in prevention and early intervention programs for BDD.

## Highlights

• Perfectionism was associated with concurrent BDD symptoms among adolescents.
• This link remained significant even when controlling for anxiety and depression.
• Perfectionism predicted changes in BDD symptoms over a 6-month period.
• BDD symptoms were specifically linked with self-oriented perfectionism.

## 1 Introduction

Body dysmorphic disorder is characterised by an intense preoccupation with perceived flaws in one's own physical appearance, which appear minimal or completely unobservable to others (APA, 2013). The disorder typically emerges during adolescence (Bjornsson et al., 2013), affecting an estimated 1.7–3.6% of young people (Möllmann, Dietel, Hunger, & Buhlmann, 2017; Schneider, Turner, Mond, & Hudson, 2016), and causes marked functional impairment in social, educational and family functioning (Albertini & Phillips, 1999; Mataix-Cols et al., 2015). Surprisingly little is known about mechanisms underpinning the development of the disorder, despite the implications such knowledge could have for the identifying vulnerable groups and informing prevention strategies.

Cognitive behavioural models propose that certain personality traits, including perfectionism, are risk factors for BDD (Veale, 2004; Wilhelm, 2006). The construct of perfectionism encompasses self-oriented perfectionism, defined as having excessively high personal standards, and socially-prescribed perfectionism, defined as a belief that others hold abundantly high standards for oneself (Hewitt & Flett, 1991). Substantial evidence indicates that perfectionism is elevated in anxiety, depression and eating disorders, with prospective studies suggesting that it may be a transdiagnostic risk factor for the development of these conditions (Egan, Wade, & Shafran, 2011). Although less empirical attention has been given to potential links between perfectionism and BDD, it has been argued that individuals with elevated levels of self-oriented perfectionism may be more likely to notice minor flaws in their appearance and to be self-critical of them (Wilhelm, 2006). Additionally, such individuals may tend to over-value physical appearance, further compounding distress caused by their own perceived appearance flaws (Blakey, Abramowitz, & Mahaffey, 2016). It has also been suggested that high levels of socially-prescribed perfectionism could lead individuals to believe that others are judging them against excessively high appearance standards, thereby increasing vulnerability to BDD (Bartsch, 2007).

To date, only a small number of studies have examined links between perfectionism and BDD. Several studies have found that individuals with BDD score higher on perfectionism measures than healthy controls (Buhlmann, Etcoff, & Wilhelm, 2008; Hartmann, Thomas, Greenberg, Matheny, & Wilhelm, 2014; Schieber, Kollei, de Zwaan, Müller, & Martin, 2013). Furthermore, perfectionism scores have been shown to be associated with BDD symptoms in non-clinical populations (Arji, Borjali, Sohrabi, & Farrokhi, 2016; Bartsch, 2007; Blakey et al., 2016). Importantly, perfectionism has been found to be a unique predictor of BDD symptoms even after controlling for co-existing psychopathology (Bartsch, 2007; Blakey et al., 2016; Hartmann et al., 2014), showing that the relationship between perfectionism and BDD exists above and beyond its link with other psychiatric symptoms.

Although there is emerging evidence for an association between perfectionism and BDD, there are several notable limitations of previous studies. First, previous studies have utilised cross-sectional designs, meaning that the direction of effects between perfectionism and BDD are unknown. Second, studies have exclusively focussed on adult samples, despite the fact that adolescence is a crucial period for the emergence of BDD symptoms (Bjornsson et al., 2013) and therefore an ideal time to investigate aetiological mechanisms. Third, to our knowledge only two studies have examined the association between BDD and different facets of perfectionism. The first found BDD symptoms to be associated with socially-prescribed but not self-oriented perfectionism among university students (Hanstock & O'Mahony, 2002). The second found both socially-prescribed and self-oriented perfectionism to be related to BDD symptoms in a university student sample, but only socially-prescribed perfectionism was elevated among those scoring above cut-off for “probable BDD” (Bartsch, 2007). While both of these studies highlight the importance of socially-prescribed perfectionism in BDD, other data suggest that BDD patients are more concerned with meeting their own aesthetic standards as opposed to meeting the expectations of others (Veale, Kinderman, Riley, & Lambrou, 2003).

The current study aimed to address gaps in the current literature, by establishing the cross-sectional and longitudinal associations between perfectionism and BDD symptoms in an adolescent population. Based on prevailing cognitive behavioural theories and previous empirical findings, we hypothesised that self-oriented and socially-prescribed perfectionism would be independently associated with BDD symptoms, even after controlling for co-existing psychopathology. In addition, we hypothesised that perfectionism would positively predict increases in BDD symptoms over time.

## 2 Methods

### 2.1 Participants

Participants were 14–16 year old students recruited from a government-funded school in south London, United Kingdom. At Time 1 (May 2017), 317 young people consented to participate. Fifteen participants were excluded from analyses because they failed complete one or more measure (*n* = 10) or their responses indicated that they had not followed instructions (e.g. reporting non-appearance related worries on the BDD questionnaire) (*n* = 5). Thus, the final sample at Time 1 consisted of 302 students (47.0% male, 52.0% female, 1.0% unknown). Six months later (November 2017), 77 participants from the original sample completed Time 2 questionnaires (response rate of 25%). Nine were subsequently excluded due to incorrect entry of participant numbers, leaving a total of 68 participants at Time 2 (27.9% male, 72.1% female). Logistic regression showed that attrition was predicted by school year group (Year 11 participants more likely to drop-out than Year 10), but no other variable (see Table A.1 in appendix).

### 2.2 Measures

#### 2.2.1 The body image questionnaire-child and adolescent version (BIQ-C; Veale, 2009)

The BIQ-C is a 12-item measure of BDD symptom severity. The questionnaire begins with a screening question and participants who report disliking at least one feature of their appearance answer 12 subsequent items. In the current study, in order to ensure that the measure captured BDD symptoms and not eating disorder psychopathology, participants were told to report on any appearance concerns *except* for worries about body weight and fatness. The BIQ-C has been shown to be internally reliable and scores discriminate between groups of adolescents with probable BDD versus no BDD (Schneider et al., 2016). Furthermore, the BIQ-C is based on, and virtually identical to, the Cosmetic Procedure Screening questionnaire which has been shown to have good test-retest reliability and convergent validity (Veale et al., 2012). In the current study, the BIQ-C was completed at both timepoints and internal consistency was excellent (α = 0.90 at Time 1; α = 0.89 at Time 2).

#### 2.2.2 The Child-Adolescent Perfectionism Scale (CAPS; (Flett et al., 2016)

The CAPS is a widely-used, 22-item self-report measure of perfectionism. It comprises two subscales measuring socially-prescribed perfectionism and self-oriented perfectionism. The CAPS has good internal consistency, test-retest reliability and concurrent and discriminant validity (Flett et al., 2016). In the current study, the CAPS was completed at Time 1. Internal consistency was good for the full scale (α = 0.90) and both subscales (α = 0.84 for self-oriented perfectionism; α = 0.88 for socially-prescribed perfectionism).

#### 2.2.3 The Revised Child Anxiety and Depression Scale – Short Version (RCADS-25 (Ebesutani et al., 2012);

The short, self-report version of the RCADS consists of 25 items assessing the presence of anxiety and depressive symptoms in youth. The RCADS-25 has good internal consistency, test-retest reliability, and convergent and divergent validity (Ebesutani et al., 2012; Kösters, Chinapaw, Zwaanswijk, van der Wal, & Koot, 2015; Piqueras, Martín-Vivar, Sandin, San Luis, & Pineda, 2017). In the current study, the RCADS-25 was completed at Time 1, and internal consistency was excellent (α = 0.92).

### 2.3 Procedure

Ethical approval for the study was granted by the Psychiatry, Nursing and Midwifery Research Ethics Subcommittee of King's College London (HR-16/17-3877). Parents were provided with written information about the study and were asked to complete a form to opt-out if they did not wish their child to participate. Adolescents were subsequently provided with a verbal and written description of the study in the classroom and asked to provide informed consent. Questionnaires were completed confidentially and participants were informed that confidentiality would only be broken if their responses indicated significant cause for concern. At Time 1 (May 2017), study questionnaires were completed in classrooms during lesson time, supervised by members of the research team and/or teachers. Most questionnaires were completed online, but where access to computers was not available, paper copies were provided. At Time 2 (November 2017), constraints in the school timetable meant that we were unable to administer questionnaires during lesson time, and therefore weblinks for the online questionnaires were sent to participants via the school's electronic homework system. Over the following month, participants were sent two follow-up messages reminding them to complete the questionnaires. In addition, they were given verbal and written reminders in school assemblies and newsletters.

### 2.4 Statistical analyses

A series of standard (i.e. simultaneous), linear regressions were used to test the association between perfectionism and BDD symptoms, both cross-sectionally and longitudinally, and with and without adjustment for anxiety and depressive symptoms. For all regression models, there were cases with high studentized residuals (i.e. outliers) and high leverage points. Cook's distances confirmed the presence of cases with high influence for all regression models (see Table A.2 in appendix). Inspection of influential cases did not reveal any reason for their exclusion. Thus, robust regression models were used to manage influential data points. This method enables the retention of influential cases when there is no clear rationale for their exclusion, but effectively weights and re-weights cases in order to minimise the impact of influential data points (Rousseeuw & Leroy, 2005). Any cases with Cook's distance greater >1 are dropped and cases with large absolute residuals are down-weighted in an iterative process, which stops when the maximum change between the weights from one iteration to the next is below tolerance. All analyses were completed using Stata version 14.2 and controlled for sex.

## 3 Results

Descriptive statistics are reported in Table 1. At Time 1, 7.3% of the sample (n = 22) scored 59 or above on the BIQ-C, suggestive of probable BDD (Veale et al., 2009). Of note, BDD symptoms significantly decreased between Time 1 and 2 for the overall group (*t*(67) = 2.24, *p* < .05), with 54% of the sample (*n* = 37) experiencing a reduction and 46% experiencing an increase (*n* = 31) in BDD symptoms. Analysis of sex effects on BDD symptoms, perfectionism and anxiety/depression revealed that females reported significant higher levels of BDD symptoms, anxiety and depression at Time 1 compared to males (see Table A.3 in the appendix). However, males and females did not differ significantly with respect to levels total perfectionism, socially-prescribed perfectionism or self-oriented perfectionism (see Table A.3). There was no difference between year groups (Year 10 versus 11) on scores of BDD, perfectionism, anxiety or depression (see Table A.4 in the appendix).

**Table 1:** Descriptive statistics for study measures.

	Mean	Standard deviation
**Time 1**		
BIQ-C	34.53	18.46
CAPS Total	64.93	15.91
SOP subscale	37.16	9.27
SPP subscale	27.77	9.06
RCADS-25	20.36	12.38
**Time 2**		
BIQ-C	31.40	17.45

***Note:*** BIQ-C = Body Image Questionnaire–Child and Adolescent Version; CAPS = Child and Adolescent Perfectionism Scale; SOP = self-oriented perfectionism; SPP = socially-prescribed perfectionism; RCADS-25 = Revised Child Anxiety and Depression Scale–Short Version.

### 3.1 Cross-sectional associations between perfectionism and BDD symptoms

The results of the cross-sectional analyses are shown in Table 2. In the first set of analyses, robust regression models were used to test the concurrent association between the total CAPS score and BIQ-C score, with and without adjustment for total RCADS-25 score. Perfectionism was found to be positively related to BDD symptoms (β = 0.38, *p* < .001). The magnitude of this association decreased when controlling for co-existing anxiety and depressive symptoms, but nevertheless remained significant (β = 0.10, *p* < .05). These analyses were then repeated using the CAPS subscale scores. Both self-oriented and socially-prescribed perfectionism were positively associated with BDD symptoms (β = 0.21, *p* < .01 and β = 0.22, *p* < .01, respectively). However, when controlling for concurrent anxiety and depression, only self-oriented perfectionism continued to predict unique variance in BDD symptom severity (β = 0.11, *p* < .05).

**Table 2:** Results of robust regression models showing cross-sectional associations between perfectionism and BDD symptom severity at Time 1.

	BDD symptom severity			
	Without adjustment for anxiety and depressive symptoms		With adjustment for anxiety and depressive symptoms	
	β	*t*	β	*t*
**Model 1**				
Factor 1: Total perfectionism	.38 (.27–.49)	6.98***	.10 (.01–.19)	2.15*
	*F* (2, 299) = 24.05; *R*^2^ = .12		*F* (3, 298) = 96.10; *R*^2^ = .38	
**Model 2**				
Factor 1: Self-oriented perfectionism	.21 (.08–.34)	3.17**	.11 (.01–.21)	2.20
Factor 2: Socially-prescribed perfectionism	.22 (.09–.34)	3.28**	-.00 (−.11–.10)	−0.04
	*F* (3, 298) = 15.97; *R*^2^ = .12		*F* (4, 297) = 72.57; *R*^2^ = .39	

***Note:*** BDD = body dysmorphic disorder; 95% confidence intervals in parentheses; all analyses controlled for sex.

*p < .05; **p < .01; ***p < .001.

### 3.2 Longitudinal associations between perfectionism and BDD symptoms

The results of the longitudinal analyses are shown in Table 3. A robust regression was used to test the extent to which BDD symptoms and total perfectionism scores at Time 1 predicted BDD symptoms at Time 2. This showed that earlier BDD symptoms predicted a significant proportion of the variance in later BDD symptoms, indicating substantial symptom stability (β = 0.53, *p* < .001). Importantly, total perfectionism at Time 1 also predicted unique variance in BDD symptoms at Time 2, when controlling for BDD symptoms at Time 1 (β = 0.22, *p* < .01). Thus, total perfectionism positively predicted *change* in BDD symptoms between Time 1 and 2. Moreover, this association remained significant even when adjusting for co-existing anxiety and depressive symptoms at Time 1 (β = 0.21, *p* < .01).

**Table 3:** Results of robust regression models predicting BDD symptom severity at Time 2 from perfectionism at Time 1.

	BDD symptom severity at Time 2			
	Without adjustment for anxiety and depressive symptoms		With adjustment for anxiety and depressive symptoms	
	β	*t*	β	*t*
**Model 1**				
Factor 1: BDD symptoms at Time 1	.53 (.38–.67)	7.08***	.49 (.30–.68)	5.21***
Factor 2: Total perfectionism at Time 1	.22 (.08–.37)	.23**	.21 (.06–.37)	2.76**
	*F* (3, 64) = 36.05; *R*^2^ = .47		*F* (4, 63) = 36.05; *R*^2^ = .47	
**Model 2**				
Factor 1: BDD symptoms at Time 1	.51 (.36–.65)	6.83***	.47 (.28–.65)	4.97***
Factor 2: Self-oriented perfectionism at Time 1	.19 (.03–.34)	2.44*	.19 (.04–.35)	2.45*
Factor 3: Socially-prescribed perfectionism at Time 1	.08 (−.07–.24)	1.05	.06 (−.10–.23)	0.77
	*F* (4, 63) = 26.6; *R*^2^ = .47		*F* (5, 63) = 21.0; *R*^2^ = .47	

***Note:*** BDD = body dysmorphic disorder; 95% confidence intervals in parentheses; all analyses controlled for sex.

*p < .05; **p < .01; ***p < .001.

When examining perfectionism subscales, robust regression indicated that self-oriented but not socially-prescribed perfectionism at Time 1 predicted BDD symptom severity at Time 2, controlling for BDD symptoms at Time 1 (β = 0.19, *p* < .05 and β = 0.08, *p* = .10, respectively). Earlier self-oriented perfectionism continued to predict change in BDD symptoms, even when controlling for anxiety and depression at Time 1 (β = 0.19, *p* < .05).

## 4 Discussion

This study tested the hypothesis that perfectionism is associated with BDD symptoms cross-sectionally and prospectively in adolescents. As expected, we found that total perfectionism was positively related to concurrent BDD symptom severity. Importantly, this relationship existed above and beyond the confounding effect of co-existing anxiety and depression. Furthermore, total perfectionism was found to predict change in BDD symptom severity over a 6-month period, even when controlling for anxiety and depressive symptoms. To our knowledge, this is the first study to show that perfectionism prospectively predicts BDD symptomatology.

Analyses of perfectionism subscales indicated that both self-oriented and socially-prescribed perfectionism were related to concurrent BDD symptoms. However, contrary to our hypothesis, only self-oriented perfectionism was significantly associated with BDD symptoms after controlling for anxiety and depression. The cross-sectional relationship between socially-prescribed perfectionism and BDD was fully accounted for by its link with anxiety and depressive symptoms. Furthermore, only self-oriented perfectionism predicted change in BDD symptoms between Time 1 and Time 2, a relationship which remained significant after adjusting for anxiety and depression. Thus, our findings suggest a specific link between self-oriented perfectionism and BDD, both cross-sectionally and longitudinally. This is in contrast to the results of two previous cross-sectional studies which have emphasised associations between socially-prescribed perfectionism and BDD symptoms in student samples (Bartsch, 2007; Hanstock & O'Mahony, 2002). Of note, although one of these studies adjusted for co-existing depressive symptoms (Bartsch, 2007), neither controlled for anxiety. The current findings are in line with data suggesting that individuals with BDD are more concerned with failure to meet their own appearance standards rather than the perceived standards of others (Veale et al., 2003). Furthermore, our results are consistent with findings in anorexia nervosa, showing a specific link between self-oriented perfectionism and body image disturbance (Castro et al., 2004; Castro-Fornieles et al., 2007).

The current findings have several theoretical and clinical implications. Our results support the notion that perfectionism, specifically self-oriented perfectionism, is a risk factor for the development of BDD (Veale, 2004). Importantly, this does not imply that individuals will BDD are striving for perfection in their appearance. Most BDD sufferers view their appearance as flawed and are seeking to blend in with the norm (Veale, 2004). However, high levels of self-oriented perfectionism may partly explain why individuals with BDD are excessively focussed on, and distressed by, perceived flaws in their appearance. If replicated, our finding that self-oriented perfectionism prospectively predicts change in BDD symptoms could indicate the value of considering perfectionism as a clinical marker for BDD vulnerability. Furthermore, targeting and reducing perfectionism could ameliorate risk for BDD. Importantly, perfectionism has been shown to be a modifiable trait, with studies demonstrating that psychological interventions such as cognitive behaviour therapy are effective in decreasing perfectionism (Riley, Lee, Cooper, Fairburn, & Shafran, 2007). Future research should test whether treatment of perfectionism reduces risk for developing BDD. In addition, studies should also investigate perfectionism as a possible maintaining factor for BDD psychopathology, for example by testing the extent to which it predicts treatment response. Such a finding could indicate the importance of directly tackling perfectionism in BDD treatment.

Limitations of this study include the focus on BDD symptoms in a healthy population as opposed to diagnosable BDD, and the reliance on single-informant data which may have inflated estimates of association. Moreover, there was a high level of participant drop-out between Time 1 and Time 2, particularly among those in Year 11 (15-16 year olds). Our high drop-out rate is likely to reflect the fact that: a) at Time 2 adolescents completed questionnaires in their own time rather than in the classroom, due to constraints in the school timetable; and b) the two timepoints fell in separate academic years and therefore some participants may have left the school. The latter may explain why attrition was particularly high among Year 11s, as a proportion of students may have left or moved schools after their Year 11 examinations (i.e. General Certificate of Secondary Education examinations). Importantly, we did not find attrition to be predicted by our variables of interest and therefore it is unlikely to have biased our results. Furthermore, selective attrition has been shown to have a minimal impact on estimates of association (Wolke et al., 2009). Nevertheless, our results should be viewed as preliminary and replication is needed.

The current findings demonstrate that perfectionism is associated with BDD symptoms in adolescents, and self-oriented perfectionism prospectively predicts change in BDD symptom severity over time. If replicated, these findings highlight the potential value of targeting perfectionism in prevention programmes for BDD.

## Funding

Georgina Krebs is funded by an MRC Clinical Research Training Fellowship (MR/N001400/1).

## Conflicts of interest

None.

## Ethical approval

All procedures performed in studies involving human participants were in accordance with the ethical standards of the institutional and/or national research committee and with the 1964 Helsinki declaration and its later amendments or comparable ethical standards.

## References

[bib1] Albertini R.S., Phillips K.A. (1999). Thirty‐three cases of body dysmorphic disorder in children and adolescents. Journal of the American Academy of Child & Adolescent Psychiatry.

[bib2] APA (2013). Diagnostic and statistical manual of mental disorders.

[bib3] Arji M., Borjali A., Sohrabi F., Farrokhi N.A. (2016). Role of perfectionism and body image in the prediction of body dysmorphic disorder symptoms.

[bib4] Bartsch D. (2007). Prevalence of body dysmorphic disorder symptoms and associated clinical features among Australian university students. Clinical Psychologist.

[bib5] Bjornsson A.S., Didie E.R., Grant J.E., Menard W., Stalker E., Phillips K.A. (2013). Age at onset and clinical correlates in body dysmorphic disorder. Comprehensive Psychiatry.

[bib6] Blakey S.M., Abramowitz J.S., Mahaffey B.L. (2016). Do obsessive beliefs predict body image disturbance?. Journal of Obsessive-Compulsive and Related Disorders.

[bib7] Buhlmann U., Etcoff N.L., Wilhelm S. (2008). Facial attractiveness ratings and perfectionism in body dysmorphic disorder and obsessive-compulsive disorder. Journal of Anxiety Disorders.

[bib8] Castro J., Gila A., Gual P., Lahortiga F., Saura B., Toro J. (2004). Perfectionism dimensions in children and adolescents with anorexia nervosa. Journal of Adolescent Health.

[bib9] Castro‐Fornieles J., Gual P., Lahortiga F., Gila A., Casulà V., Fuhrmann C., Toro J. (2007). Self‐oriented perfectionism in eating disorders. International Journal of Eating Disorders.

[bib10] Ebesutani C., Reise S.P., Chorpita B.F., Ale C., Regan J., Young J., Weisz J.R. (2012). The Revised Child Anxiety and Depression Scale-Short Version: Scale reduction via exploratory bifactor modeling of the broad anxiety factor. Psychological Assessment.

[bib11] Egan S.J., Wade T.D., Shafran R. (2011). Perfectionism as a transdiagnostic process: A clinical review. Clinical Psychology Review.

[bib12] Flett G.L., Hewitt P.L., Besser A., Su C., Vaillancourt T., Boucher D., Gale O. (2016). The Child–Adolescent Perfectionism Scale: Development, psychometric properties, and associations with stress, distress, and psychiatric symptoms. Journal of Psychoeducational Assessment.

[bib13] Hanstock T.L., O'Mahony J.F. (2002). Perfectionism, acne and appearance concerns. Personality and Individual Differences.

[bib14] Hartmann A.S., Thomas J.J., Greenberg J.L., Matheny N.L., Wilhelm S. (2014). A comparison of self-esteem and perfectionism in anorexia nervosa and body dysmorphic disorder. The Journal of Nervous and Mental Disease.

[bib15] Hewitt P.L., Flett G.L. (1991). Perfectionism in the self and social contexts: Conceptualization, assessment, and association with psychopathology. Journal of Personality and Social Psychology.

[bib16] Kösters M.P., Chinapaw M.J., Zwaanswijk M., van der Wal M.F., Koot H.M. (2015). Structure, reliability, and validity of the revised child anxiety and depression scale (RCADS) in a multi-ethnic urban sample of Dutch children. BMC Psychiatry.

[bib17] Mataix-Cols D., Fernández de la Cruz L., Isomura K., Anson M., Turner C., Monzani B., Krebs G. (2015). A pilot randomized controlled trial of cognitive-behavioral therapy for adolescents with body dysmorphic disorder. Journal of the American Academy of Child & Adolescent Psychiatry.

[bib18] Möllmann A., Dietel F.A., Hunger A., Buhlmann U. (2017). Prevalence of body dysmorphic disorder and associated features in German adolescents: A self-report survey. Psychiatry Research.

[bib19] Piqueras J.A., Martín-Vivar M., Sandin B., San Luis C., Pineda D. (2017). The revised child anxiety and depression scale: A systematic review and reliability generalization meta-analysis. Journal of Affective Disorders.

[bib20] Riley C., Lee M., Cooper Z., Fairburn C.G., Shafran R. (2007). A randomised controlled trial of cognitive-behaviour therapy for clinical perfectionism: A preliminary study. Behaviour Research and Therapy.

[bib21] Rousseeuw P.J., Leroy A.M. (2005).

[bib22] Schieber K., Kollei I., de Zwaan M., Müller A., Martin A. (2013). Personality traits as vulnerability factors in body dysmorphic disorder. Psychiatry Research.

[bib23] Schneider S.C., Turner C.M., Mond J., Hudson J.L. (2016). Prevalence and correlates of body dysmorphic disorder in a community sample of adolescents. Australian and New Zealand Journal of Psychiatry.

[bib24] Veale D. (2004). Advances in a cognitive behavioural model of body dysmorphic disorder. Body Image.

[bib25] Veale D. (2009). Body image questionnaire – child and adolescent version. http://www.kcl.ac.uk/ioppn/depts/psychology/research/ResearchGroupings/CADAT/Research/Body-Image-Questionnaires.aspx.

[bib26] Veale D., Ellison N., Werner T.G., Dodhia R., Serfaty M., Clarke A. (2012). Development of a cosmetic procedure screening questionnaire (COPS) for body dysmorphic disorder. Journal of Plastic, Reconstructive & Aesthetic Surgery.

[bib27] Veale D., Kinderman P., Riley S., Lambrou C. (2003). Self‐discrepancy in body dysmorphic disorder. British Journal of Clinical Psychology.

[bib28] Wilhelm S. (2006). Feeling good about the way you look: A program for overcoming body image problems.

[bib29] Wolke D., Waylen A., Samara M., Steer C., Goodman R., Ford T. (2009). Selective drop-out in longitudinal studies and non-biased prediction of behaviour disorders. The British Journal of Psychiatry.

