# Supplementary material for: Is perfectionism a risk factor for adolescent body dysmorphic symptoms? Evidence for a prospective association
Source: J Obsessive Compuls Relat Disord. 2019 Jul;22:100445. doi: 10.1016/j.jocrd.2019.100445 (PMC6737990; doi:10.1016/j.jocrd.2019.100445)
Supplement: Multimedia component 2 [file mmc2.docx]

**Table A.1: Results of logistic regression showing variables predicting participant drop-out between Time 1 and Time 2**

| Variable | Odds Ratio  (95% confidence intervals) | *p* |
| --- | --- | --- |
| Sex | 1.00 (.98 - 1.02) | .67 |
| Year group | 0.32 (0.17 - 0.63) | <.01 |
| BIQ-C total at Time 1 | 1.01 (.99 - 1.03) | .36 |
| CAPS total at Time 1 | 1.01 (.98 - 1.03) | .69 |
| RCADS-25 total at Time 1 | 1.02 (0.99 – 1.06) | .12 |

Note: BIQ-C = Body Image Questionnaire–Child and Adolescent Version; CAPS = Child and Adolescent Perfectionism Scale; RCADS-25 = Revised Child Anxiety and Depression Scale–Short Version.

**Table A.2: Cook’s Distances for possible influential data points in each regression model (i.e. cases with Cook’s distance > 4/n)**

| Cross-sectional regressions | | | | Prospective regressions | | | |
| --- | --- | --- | --- | --- | --- | --- | --- |
| CAPS total | | CAPS subscales | | CAPS total | | CAPS subscales | |
| Without RCADS | With RCADS | Without RCADS | With RCADS | Without RCADS | With RCADS | Without RCADS | With RCADS |
| .01 | .04 | .02 | .04 | .29 | .26 | .28 | .22 |
| .44 | .02 | .02 | .02 | .07 | .10 | .14 | .09 |
| .07 | .02 | .02 | .02 | .17 | .08 | .19 | .06 |
| .04 | .04 | .33 | .03 | .22 | .45 |  | .36 |
| .03 | .02 | .05 | .03 |  | .22 |  | .21 |
| .03 | .04 | .03 | .02 |  |  |  |  |
| .02 | .02 | .02 | .03 |  |  |  |  |
| .03 | .02 | .02 | .03 |  |  |  |  |
| .02 | .01 | .05 | .02 |  |  |  |  |
| .02 | .04 | .02 | .06 |  |  |  |  |
| .13 | .08 | .02 | .07 |  |  |  |  |
|  | .02 | .01 | .02 |  |  |  |  |
|  | .02 | .02 | .03 |  |  |  |  |
|  | .04 | .02 | .05 |  |  |  |  |
|  | .05 | .02 | .02 |  |  |  |  |
|  | .02 | .05 | .03 |  |  |  |  |
|  | .03 | .10 | .02 |  |  |  |  |
|  | .01 |  | .02 |  |  |  |  |
|  | .01 |  |  |  |  |  |  |

**Table A.3: Sex effects on variables**

|  | **Males** | **Females** | **Statistical comparison** |
| --- | --- | --- | --- |
| BIQ-C | 22.25 (17.36) | 34.07 (16.57) | *t*(297) = 6.02; *p* < .001 |
| CAPS total | 61.62 (15.67) | 61.92 (16.37) | *t*(297) = .16; *p* = .87 |
| CAPS SPP subscale | 26.65 (9.13) | 25.89 (9.06) | *t*(297) = -.72; *p* = .47 |
| CAPS SOP subscale | 34.97(8.83) | 36.03 (9.27) | *t*(297) = 1.01; *p* = .31 |
| RCADS anxiety subscale | 7.14 (6.76) | 11.13 (6.92) | *t*(297) = 5.03; *p* < .001 |
| RCADS depression subscale | 6.59 (5.51) | 8.06 (5.27) | *t*(297) = 2.34; *p* < .05 |

**Table A.4: Effects on year group on variables**

|  | **Year 10** | **Year 11** | **Statistical comparison** |
| --- | --- | --- | --- |
| BIQ-C | 29.30 (19.02) | 27.21 (15.75) | *t*(300) = 0.99; *p* = .33 |
| CAPS total | 61.36 (15.90) | 62.10 (16.43) | *t*(300) = 0.99; *p* = .38 |
| CAPS SPP subscale | 25.64 (9.29) | 27.11 (8.70) | *t*(300) = 1.36; *p* = .17 |
| CAPS SOP subscale | 35.71 (8.74) | 34.99 (9.70) | *t*(300) = 0.68; *p* = .50 |
| RCADS anxiety subscale | 9.46 (7.54) | 8.89 (6.33) | *t*(300) = 0.67; *p* = .50 |
| RCADS depression subscale | 7.25 (5.77) | 7.61 (4.76) | *t*(300) = 0.56; *p* = .58 |
